# Rab7a and Mitophagosome Formation

**DOI:** 10.3390/cells8030224

**Published:** 2019-03-08

**Authors:** Esther Hui Na Tan, Bor Luen Tang

**Affiliations:** 1 Department of Biochemistry, Yong Loo Lin School of Medicine, National University of Singapore, Singapore 117597, Singapore; e0145854@u.nus.edu; 2 NUS Graduate School for Integrative Sciences and Engineering, National University of Singapore, Singapore 119077, Singapore

**Keywords:** autophagy, mitophagy, mitophagosome, Rab7, Tre-2/Bub2/Cdc16 (TBC)1D5, TBC1D15/17, TRAF family-associated NF-κB activator binding kinase 1 (TBK1)

## Abstract

The small GTPase, Rab7a, and the regulators of its GDP/GTP-binding status were shown to have roles in both endocytic membrane traffic and autophagy. Classically known to regulate endosomal retrograde transport and late endosome-lysosome fusion, earlier work has indicated a role for Rab7a in autophagosome-lysosome fusion as well as autolysosome maturation. However, as suggested by recent findings on PTEN-induced kinase 1 (PINK1)-Parkin-mediated mitophagy, Rab7a and its regulators are critical for the correct targeting of Atg9a-bearing vesicles to effect autophagosome formation around damaged mitochondria. This mitophagosome formation role for Rab7a is dependent on an intact Rab cycling process mediated by the Rab7a-specific guanine nucleotide exchange factor (GEF) and GTPase activating proteins (GAPs). Rab7a activity in this regard is also dependent on the retromer complex, as well as phosphorylation by the TRAF family-associated NF-κB activator binding kinase 1 (TBK1). Here, we discuss these recent findings and broadened perspectives on the role of the Rab7a network in PINK1-Parkin mediated mitophagy.

## 1. Introduction

Macroautophagy (or commonly abbreviated as ‘autophagy’) is a conserved cellular process in eukaryotes that involves membrane remodeling for the degradation and recycling of cellular components, be it soluble cytosolic components, cytoplasmic aggregates, or membranous organelles. The process begins with the formation of a double-membraned autophagosome [1], whose content is eventually degraded via fusion with the lysosome [2]. Autophagosome formation and maturation are primarily dependent on the action of several protein complexes, components of which were first genetically defined in *S. cerevisiae* as a large set of autophagy (*Atg*) genes, most of which have conserved orthologues in mammals. The Unc51-like kinase 1 (ULK1)/Atg1-containing complex is the upstream component of physiological autophagy signaling, taking cues from the nutrient-sensing and stress response pathways regulated by the molecular target of rapamycin (mTOR) complex 1 (mTORC1) and liver kinase B1 (LKB1)-AMP-activated protein kinase (AMPK). ULK1 phosphorylates Beclin1/Atg6 and activates the second complex containing Beclin1 and the phosphatidylinositol 3-kinase Vps34 [3], whose activity results in localized production of phosphatidylinositol (3,4,5)-trisphosphate. The latter serves as a docking platform for the recruitment of components required for the formation of an autophagy specific initiating structure known as the phagophore or the isolation membrane (IM), such as the WD-repeat protein interacting with phosphoinositides (WIPI) family members/Atg18 [4]. Upon initiation, the phagophore/IM then further expands to form an autophagosome with the enclosure of the cytoplasmic contents. Expansion and eventual membrane closure of the phagophore/IM to form a free autophagosome is known to be dependent on the formation of phosphoethanolamine (PE)-lipidated microtubule-associated protein light chain 3 (LC3) (or other Atg8/LC3/Gamma-aminobutyric acid receptor-associated protein (GABARAP) family members). LC3 is first processed at its C terminus by Atg4 to LC3-I, and LC3-I is subsequently conjugated with PE to LC3-II by the Atg16L complex, which acts like an E3 ubiquitin ligase [5]. LC3 (and its lipidated form) is an established autophagosome marker, and together with ubiquitin-binding adaptor proteins or autophagy receptors, such as sequestosome-1/p62, plays important roles in the selective capture of autophagic cargo. However, there is also evidence to suggest that the LC3/GABARAP proteins are more critical for autophagosome-lysosome fusion rather than autophagosome formation [6]. 

Mammalian cells appear to have multiple potential subcellular sites for nascent autophagosome formation, including membrane organelle intersections, such as the trans-Golgi network (TGN) [7], endoplasmic reticulum (ER)-mitochondria contact sites [8], ER exit site [9], ER-plasma membrane contact sites [10] as well as Rab11-positive recycling endosomes [11]. The most prominent membrane sources for the expansion of the nascent autophagosome include ER-derived Coat protein II (COPII) vesicles [12,13,14,15] and TGN-endosome derived Atg9a-containing vesicles [16,17]. Of note, Atg9 is the only known membrane-spanning protein among all the essential autophagy proteins. Typically localizing at the membrane of the TGN and recycling endosomes as well as a subpopulation of cytoplasmic membrane vesicles, Atg9 is translocated to the sites of the autophagosome biogenesis upon autophagy induction, with its phagophore targeting dynamics regulated by ULK1 and Src phosphorylation [18,19]. Forming a complex with Atg2 and Atg18, the exact role(s) of Atg9 in autophagosome biogenesis per se has remained poorly understood, although recent work suggests that one of the functions of this complex is to establish contact sites between the ER and phagophores [20]. 

In the more specific case of organelle autophagy involving damaged mitochondria, or mitophagy, a well-known mechanism of action is dependent on two gene products, mutations of which predispose an individual to familial Parkinson’s disease (PD), namely PTEN-induced kinase 1 (PINK1) protein kinase and the Parkin E3 ubiquitin ligase [21,22]. Normally targeted to the mitochondria, PINK1 is first imported and then rapidly degraded. Upon mitochondrial damage, resulting in a depolarization of the mitochondrial membrane potential that is required for import, PINK1 becomes stabilized on the mitochondrial outer membrane [23,24], where it can phosphorylate ubiquitin as well as the ubiquitin-like domain of Parkin. PINK1′s recruitment of Parkin to the mitochondrial outer membrane and the latter’s activation result in a cumulative ubiquitination and the formation of ubiquitin chains on multiple proteins. PINK1 and the ubiquitin signal generated by Parkin recruits autophagy receptors, particularly nuclear domain 10 protein of 52 kDa (NDP52) and optineurin (OPTN) [25], which binds to both ubiquitin chains and LC3 (via an LC3 interaction region (LIR) motif), thus facilitating the engagement of mitophagosome formation sites, such as the ER-mitochondria contacts [26]. Other than being an important membrane source during the early steps of autophagosome formation [15], Atg9-containing vesicles are also targeted to the depolarized mitochondria at the initial stages of PINK1-Parkin-mediated mitophagy [27].

The autophagy processes and mechanisms intersect with, and are clearly dependent on, vesicular membrane trafficking machinery for the delivery of membrane materials to the site of phagophore formation or nascent autophagosome maturation, as well as for autophagosome-lysosome fusion. Autophagosome-lysosome fusion is critically dependent on specific soluble NSF attachment protein receptors (SNAREs) [28], particularly the autophagosome targeted syntaxin 17 (Stx17) [29], as well as longin SNARE Ykt6 [30,31,32,33,34]. In this regard, multiple members of the Rab family of small GTPases, with classical roles in exocytic and endocytic membrane trafficking, are also known to be important or critical for autophagy [35,36,37]. Rab GTPases are molecular switches that regulate vesicular membrane traffic in eukaryotes by regulating the toggling between their GDP versus GTP bound states [38,39]. Rab guanine nucleotide exchange factors (GEFs) activate Rabs by catalyzing the GDP–GTP exchange while GTPase activating proteins (GAPs) promote Rab GTP hydrolysis, which inactivates the latter [40]. GTP-bound activated Rabs engage effector molecules in triggering downstream membrane trafficking events [41], and a cascade of Rab GEF and GAP actions [42] could provide a directional handover of one Rab-regulated step to another in the flow of membrane traffic. 

Rabs that are particularly prominently implicated in autophagosome formation include Rab1/Ypt1 [43,44,45], Rab32 [46], and Rab33B [47]. On the other hand, autophagosome fusion with the lysosome is often attributed to the activity of the endolysosomal Rab, Rab7a [48,49]. Rab35 is another known regulator of autophagy, which acts through its engagement of the autophagy receptor, NDP52 [50]. Interestingly, however, recent studies on PINK1-Parkin-mediated mitophagy have suggested that Rab7a also has a role in mitophagosome formation. These works and the new perspectives formed are discussed in the paragraphs below. 

## 2. Rab7a’s Implicated Roles in Autophagosome-Lysosome Fusion

We shall first take a quick look at the small GTPase in question and what is previously known about its role in autophagy. Yeast Ypt7p and the mammalian paralogues, Rab7A and Rab7B, are key regulators of endosomal membrane traffic. Rab7A and Rab7B share only about 50% homology and these have documented differences in their function in terms of endosomal traffic [51]. In fact, a recent finding suggests that Rab7b is a negative regulator of autophagy and that it regulates LC3 processing by modulating Atg4B activity [52]. The generic term, Rab7, sometimes used in the discussions here, unless otherwise specified, would therefore pertain to Rab7a. Rab5 and Rab7a are two key regulators of endosomal traffic, thus forming the earliest steps of endocytosis to lysosomal targeting and degradation. Along this pathway, a process of “Rab conversion” occurs during early to late endosomal transport (or early to late endosomal maturation), involving a succession of Rab5 by Rab7a [53,54]. Rab7a is activated and recruited to the late endosome by the heterodimeric Mon1–Ccz1 complex, which is a Rab5 effector as well as Rab7a GEF [55,56]. A critical Rab7a effector in endo-lysosomal transport and fusion is the Rab7a-interacting lysosomal protein (RILP) [57], whose engagement of dynein-dynactin motor complexes to Rab7a-containing late endosomes and lysosomes effectively inhibits peripheral dispersion and clusters these membranes at the perinuclear region [58]. Another important effector of Rab7 is the retromer complex, which functions in retrograde endosomal transport [59,60]. The activation of the roles and mechanisms of Rab7 in endosomal and endo-lysosomal transport were extensively reviewed recently [48,49], and shall not be further elaborated here.

Rab7a’s role in endolysosomal transport/fusion suggests that it could potentially also regulate autophagosome–lysosome fusion, which has indeed been fairly extensively documented [61,62,63,64]. In particular, silencing of Rab7a causes an accumulation of late autophagic vacuoles, indicating a need for Rab7a’s activity in their destruction by fusion with lysosomes [61,62]. Mechanistically, the activation of Rab7 in the context of autophagy was shown in yeast and invertebrate models to also utilize the known Rab7a GEF, monensin sensitivity 1-Calcium-caffeine-zinc sensitivity protein 1 (Mon1–Ccz1). Loss of the Ccz1-Mon1-Rab7 module resulted in an accumulation of autophagosomes in starved *Drosophila* fat cells due to impaired autophagosome–lysosome fusion [63]. Ccz1-Mon1 recruits Ypt7/Rab7 to autophagosomes, and in yeast, this is shown to occur through the GEF’s direct binding to Atg8 via the LC3 interacting region of Ccz1 [65]. 

Activated Rab7a engages effector molecules that could promote autophagosome–lysosome fusion in complementary ways. The FYVE and coiled-coil (CC) domain-containing 1 (FYCO1) adaptor protein is a Rab7a effector that facilitates kinesin-driven movement towards the cell periphery [66], whereas RILP facilitates the recruitment of dynein-dynactin motor complexes for movement towards perinuclear regions [58]. Another Rab7a effector, the cholesterol sensing oxysterol-binding protein-related protein 1L (ORPL1), plays a role in the formation of ER-autophagosome contacts [67]. The Rab7a effector, pleckstrin homology domain containing protein family member 1 (PLEKHM1), interacts with the homotypic fusion and protein sorting (HOPS) complex, which is the tethering complex that aids Stx17-mediated autophagosome-lysosome fusion [68,69] and has an LIR that mediates its binding to autophagosomal membranes [70]. Yet another Rab7 effector, the ectopic P-Granules autophagy protein 5 homolog (EPG5), stabilizes and facilitates the assembly of the STX17-SNAP29-VAMP7/8 trans-SNARE complexes between autophagosomes and lysosomes, which drives fusion [64]. A role for Rab7 GAPs in autophagy is less well-defined. However, the Rab7 GAP, Armus/Tre-2/Bub2/Cdc16 (TBC)1D2, which inactivates Rab7a, is recruited to autophagosomes via interactions with LC3 [71] and is important for proper autophagic flux [72]. A very comprehensive update on our current understanding of autophagosome–lysosome fusion was recently provided by Nakamura and Yoshimori [73], and the reader is referred to their excellent commentary for further details. A recent finding by Kuchitsu and colleagues, however, suggested that unlike the case in yeast and *Drosophila*, Rab7 is required for autolysosome maturation and not autophagosome–lysosome fusion in mammalian cells [74,75]. 

## 3. Rab7a’s Role in Mitophagy—Rab7a GEF, GAPs, and Effectors Mediate Rab7a Targeting and Activity at the Damaged Mitochondria

An early hint that Rab7 could also play a role in certain specific types of autophagosome biogenesis came from its apparent role in the formation of group A streptococcus (GAS)-containing autophagosome-like vacuoles (GcAVs) [76]. GAS could enter cells via the endocytic pathway, but upon escape from the endosomal membrane, the bacteria could be captured by autophagosome-like compartments that would facilitate their destruction via fusion with lysosomes [77]. In infected cells, small, LC3-decorated vesicles sequester the Streptococcal chains, and these coalesce into a single, large GcAV. The small GcAV formation is disrupted by Rab7 silencing or the expression of a dominant negative Rab7 mutant, thus attesting to a specific requirement for the small GTPase in GcAV biogenesis [76].

The first clear indication that Rab7 could play a role in the early stages of mitophagy came from the findings of Yamano and colleagues [78]. The authors examined a specific Rab7a GAP TBC1D15, which is mitochondria-localized via its interaction with the mitochondrial outer membrane protein, fission 1 (FIS1), where it plays a role in regulating mitochondrial morphology [79] and mitophagy [80,81]. Like Armus/TBC1D2, TBC1D15 associates with both the mitochondria (through the binding of mitochondrial Fis1) and the autophagic IM (by interacting with the LC3/GABARAP/Atg8 family proteins). Interestingly, during Parkin-mediated mitophagy induced by the ionophore valinomycin, *TBC1D15-/-* cells as well as *FIS1-/-* cells exhibited an excessive accumulation of LC3 signals and tubules decorated with LC3. The loss of the Rab7a GAP TBC1D15 therefore appears to result in excessive proliferation of autophagosomal structures around damaged mitochondria. This phenotype is dependent on the Rab GAP activity of TBC1D15. As excessive autophagosome formation is not an obvious phenotype in starvation-induced autophagy, thus this appears to be specific for the process of mitophagy. Silencing of Rab7 suppressed the abnormal LC3 accumulation and membrane tubulation in both *TBC1D15-/-* and *FIS1-/-* cells. Rab7 is targeted to the phagophore/IM upon mitophagy induction, and is also found on the LC3-decorated tubules. TBC1D17, which is homologous to TBC1D15, also binds FIS1 and participates in mitophagy by forming homodimers and heterodimers with TBC1D15, and its loss likewise produced a similar LC3 phenotype. Although not previously known to be a Rab7a GAP, the loss of the TBC1D17-induced phenotype was suppressed by Rab7a silencing. It therefore appears that these two mitochondria-targeted GAPs moderate a Rab7a activity that is important for mitophagosome formation or biogenesis, rather than its destruction. 

An important notion arising from the above findings is that Rab7a is targeted to the mitochondria during mitophagy. However, Rab7a is best known to be localized to the late endosome/lysosomes membranes. How then is it able to function at the mitochondria? More recent work by Yamano and colleagues [82] showed that in valinomycin-induced mitophagy, RAB7a is indeed recruited to damaged mitochondria. This recruitment is affected by the MON1/CCZ1 RAB7a GEF complex, as the silencing of these prevented RAB7a mitochondria targeting. In fact, not only could MON1-CCZ1 and RAB7a be recruited to the mitochondria, two other key components of the Rab module upstream of RAB7a in the endosomal traffic, namely RAB5 and its GEF, RABGEF1/RABEX-5 [83], are also recruited to damaged mitochondria. RABGEF1′s mitochondrial recruitment is dependent on Parkin activity, and the former binds to ubiquitin chains via its ubiquitin binding domains [84,85]. As per the endosomal Rab5-Rab7 switch mentioned earlier for endo-lysosomal membranes, MON1/CCZ1 as a RAB5 effector [54] could thus be recruited by the RABGEF1-activated RAB5 to damaged mitochondria, with the former in turn recruiting RAB7a.

Another report by Jimenez-Orgaz et al. [86] provided a different mechanistic perspective on how Rab7′s subcellular localization and activity in mitophagy is regulated, namely via a Rab7a effector, the retromer complex, as well as another Rab7a GAP, TBC1D5. Previous work by others have shown that membrane recruitment of the retromer complex is catalyzed by RAB7a and inhibited by TBC1D5 [60]. Using an antibody that was able to detect endogenous RAB7a in HeLa cells by immunofluorescence microscopy, the authors found that other than the endolysosomal membranes, a substantial amount of endogenous RAB7a could be found colocalizing with the markers of mitochondria, and the ER and TGN membranes. Loss of the retromer subunits, VPS29 or VPS35, resulted in enhanced RAB7a localization to LAMP2-decorated late endosomal-lysosomal membranes and hyperactivation of the small GTPase. The enhanced endo-lysosomal localization of RAB7a could be due to its increased engagement with its lysosome effector, RILP. Rab7 activity controlled by the retromer was in line with the observation that neither an inactive RAB7a-T22N mutant nor a constitutively active RAB7-Q67L mutant demonstrated any significant subcellular localization shifts with the loss of the retromer. 

Interestingly, the RAB7a localization shift phenotype associated with a loss of retromer function is phenocopied by a loss of the RAB7a GAP TBC1D5, which was known earlier to bind to the retromer subunit, VPS29 [87]. A VPS29-L152E mutant that could no longer bind TBC1D5 could not rescue the RAB7a localization shift phenotype in VPS29-deficient cells, although it was able to restore the stability of VPS35, which is otherwise readily degraded in the absence of VPS29. TBC1D5 deficiency also resulted in heightened RAB7a activity, and this could be reversed by over-expression of the wild-type protein, but not a VPS29 binding mutant. Importantly, retromer and TBC1D5′s regulation of RAB7a localization and activity has little to do with retromer-dependent recycling of receptor molecules, but are instead required for mitophagy [86]. The effect of retromer loss on starvation-induced autophagy was minor, and retromer-TBC1D5 regulated RAB7a activity is thus not likely to be critical in this process. However, mitochondrial clearance in HeLa cells over-expressing Parkin and treated with the proton uncoupler, Carbonyl cyanide m-chlorophenyl hydrazone (CCCP), were significantly impaired in VPS29-, VPS35-, and TBC1D5-deficient cells. This impairment could be rescued by the over-expression of the corresponding wild-type proteins, but not by mutants defective in the VPS29–TBC1D5 interaction. The importance of the retromer complex in mitophagy is also illustrated by another cell model, SH-SY5Y, which expresses endogenous Parkin and a mitochondria-anchored pH-sensitive mCherry sensor construct to assay for lysosomal delivery. In this model, lysosomal delivery of damaged mitochondria in VPS35-silenced cells was indeed significantly lost. 

How does the retromer and TBC1D5 affect mitophagy? It is conceivable that the shift of RAB7a to the endo-lysosomal membranes with a loss of either the retromer or TBC1D5 could prevent its targeting to damaged mitochondria. Indeed, cells undergoing mitophagy had RAB7a-positive structures covering or surrounding the Parkin- or translocase of outer membrane 20 (TOM20)-labeled damaged mitochondrial clusters. However, RAB7a could not be detected on the damaged mitochondria in VPS29 or VPS35 deficient cells. Also, unlike wild-type RAB7a, the constitutively active RAB7-Q67L is impaired in terms of localization to damaged mitochondria after CCCP treatment. Knockout of RAB7a led to a pronounced impairment in mitochondrial clearance, which is fully rescued by wild-type RAB7a, but not by RAB7a-Q67L. These findings indicated that retromer-TBC1D5 could either maintain a fraction of RAB7a at the mitochondrial membrane, or aids in the latter’s targeting of damaged mitochondria. In either case, RAB7a’s guanine nucleotide cycling between the GDP/GTP-bound states at the mitochondria is important for efficient mitophagy. The constitutively active RAB7-Q67L could not be efficiently targeted to the damaged mitochondria, suggesting that RAB7a targeting is dependent on the RAB7a GEF MON1-CCZ1, which prefers to bind to GDP-bound RAB7a.

Taken together, the findings discussed above indicate that Rab7a could be targeted to the damaged mitochondria during mitophagy induction and functions via the aid of its GEF, effector, and GAPs. Furthermore, controlled Rab7s GDP-GTP exchange, as well as its subsequent GTP hydrolysis, are critical for the efficient initiation of mitophagy. Importantly, Rab7a and its activity appears to be required early, i.e., at the mitophagosome formation stage, well before the previously documented need for its activity at the late stage of mitophagosome–lysosome fusion. It is also worth noting that in the reports discussed above, specific Rab7a recruitment and its activity may not be important for autophagosome formation in the more general context of starvation induced autophagy, but appears to be critical for mitophagy to eliminate damaged mitochondria. 

## 4. Rab7a Is Important for Mitophagosome Formation by Regulating the Phagophore Transport of Atg9a-Containing Vesicles

Why is the activity of Rab7 required during mitophagosome formation, and how does Rab7 drive mitophagosome formation? In examining autophagy structures around valinomycin treatment-damaged mitochondria, Yamano and colleagues [82] noted two important clues. Firstly, numerous LC3-decorated spherical structures surrounding the mitochondria during mitophagy, with the latter marked by the matrix marker, pyruvate dehydrogenase E1 alpha 1 (PDHA1). Silencing of RAB7a did not significantly impair LC3 recruitment (manifested as spotty structures) to damaged mitochondria, but the number of LC3-labelled autophagosomes containing PDHA1 was significantly reduced. It would seem that the LC3-decorated phagophores in RAB7A-depleted cells could not adequately enclose damaged mitochondria. Secondly, RAB7A silencing resulted in an apparent impairment in the recruitment of ATG9A. ATG9A is typically found at the golgi/TGN and small vesicular structures in the cytosol, and ATG9A-bearing vesicles or membranes are known to be an important membrane source for autophagosome formation [16]. Upon mitophagy stimulation, ATG9A would typically assemble into larger spots on the mitochondria, but this assembly is impaired in the absence of RAB7a. The ATG9A signal at the Golgi/TGN is visually reduced upon mitophagy induction, but this reduction is also diminished by the loss of RAB7a. Upon mitophagy induction, ATG9A-positive structures become colocalized with LC3-labeled autophagic membranes. This colocalization is significantly enhanced in TBC1D15/17 double-knockout cells. These observations, taken together, suggest that phagophore expansion during mitophagy is dependent on an input from ATG9A-bearing membranes, and that this input is significantly diminished in the absence of RAB7a.

Jimenez-Orgaz and colleagues made similar observations in their system [86]. After mitophagy induction with CCCP in HeLa cells, ATG9a from the TGN and cytosolic vesicular structures translocate to Parkin-decorated damaged mitochondria. This translocation is impaired in retromer-deficient cells. Appropriate spatial control of RAB7a activity within the cell appears to be necessary for ATG9a translocation and mitophagosome formation. RAB7a was known to exhibit a certain degree of colocalization with ATG9a. In VPS35 knockout cells, however, there is a significant loss of co-localization between RAB7a and endogenous ATG9a, particularly at the TGN region (both before and after CCCP-treatment) as RAB7a is sequestered by the lysosomes. In a clonal RAB7a-knockout cell line expressing RAB7a-Q67L, much less endogenous LC3 was observed around damaged mitochondria after CCCP treatment. Mitophagosome formation is therefore defective in cells expressing only RAB7a that is not subjectable to proper guanine nucleotide cycling regulation, which appears to be required for ATG9a membrane recruitment to the growing phagophore around the damaged mitochondria.

**Figure 1:**
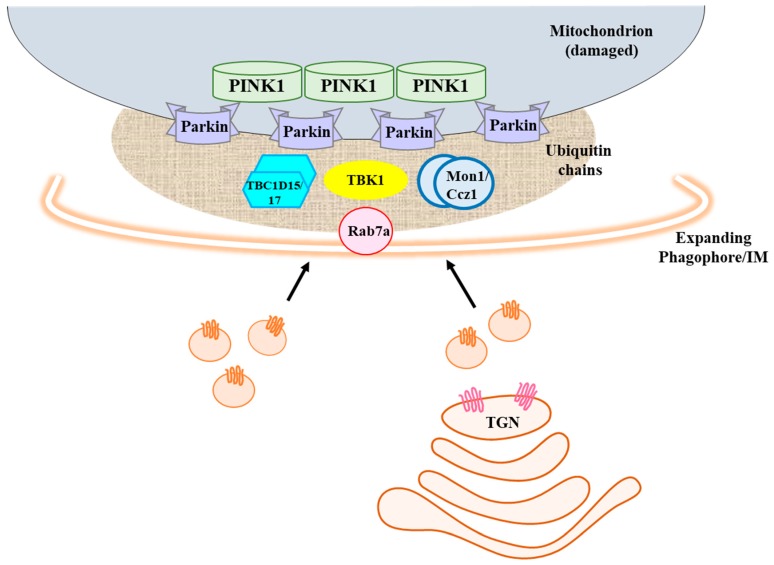
A schematic diagram depicting a role for Rab7a at the initial stage of mitophagy. Damaged mitochondria become coated with ubiquitin chains, proliferated on its outer membrane proteins by Parkin, which is recruited and activated by PINK1 stabilized on the mitochondrial surface. Rab7a is also recruited to damaged mitochondria, likely by its GEF Mon1-Ccz1. Rab7a’s activity at the mitochondria is dependent on proper cycling of its guanine nucleotide binding effected by its GEF and GAPs, like TBC1D15/17 and TBC1D5 (not shown here for brevity), and is also modulated by TBK1 phosphorylation. Rab7a at the damaged mitochondria facilitates the input of Atg9a containing membranes into the growing phagophore. See text for more details.

The findings discussed above suggest that Rab7a recruited to damaged mitochondria is important for the regulation of an input of Atg9a containing membranes for phagophore expansion around damaged mitochondria (schematically summarized in Figure 1). How this occurs is currently uncertain. Trafficking of Atg9a and its associated membranes in autophagy has been linked to adapter protein 4 (AP4) complex [88] as well as Rab1 and its GEF, the transport protein particle (TRAPP)III complex [89], as well as the autophagy-regulating TBC1D14 [90,91], but not directly to Rab7a. Understanding how Rab7a modulates Atg9a traffic and phagophore expansion would therefore require more work. 

## 5. Mitophagy Modulation by Rab7a Phosphorylation

Beyond the typical geranylgeranylation with a lipid anchor, Rab7a’s activity is also regulated by a number of other post-translational modifications [92]. Rab7a undergoes serine-threonine phosphorylation at S72 as well as tyrosine phosphorylation at Y183 by Src kinase [93]. Interestingly, the phosphate group at Rab7 S72 could be dephosphorylated by phosphatase and tensin homolog (PTEN) [94], and isoforms of PTEN were recently shown to differentially regulate mitophagy [95,96]. In this regard, an interesting recent report by Heo et al. has now identified a kinase for Rab7 S72 and provided further understanding of Rab7′s role in mitophagosome formation [97]. 

Heo and colleagues have previously found that the assembly of ubiquitin chains on mitochondria triggers the recruitment of autophagy receptors and the activation of the TRAF family-associated NF-κB activator binding kinase 1 (TBK1). The latter phosphorylates these receptors, including OPTN, NDP52 [98], and SQSTM1/p62 [99]. In a new phosphoproteomic screen, the authors found RAB7a S72 to be a novel candidate target of TBK1. TBK1 is a member of the IκB kinase (IKK) family, which has known functions in innate immunity signaling pathways and autophagy, and its mutations underlie some cases of familial amyotrophic lateral sclerosis (fALS) [100]. The residue, S72, is located at RAB7a’s “switch II” region, which is involved in guanine nucleotide exchange and effector protein interactions. Notably, a good number of Rab family members also bear an S/T residue at this corresponding position. The leucine-rich repeat protein 2 (LRRK2) protein kinase, encoded by a major susceptibility gene mutated in juvenile onset Parkinson’s disease [101], phosphorylates a distinct set of RABs in this analogous position and regulates their functions [102,103,104,105]. Heo and colleagues showed that while recombinant TBK1 directly phosphorylates S72 of RAB7a, other RABs known to be phosphorylated by LRRK2 are not its substrate, at least in vitro. Importantly, activation of TBK1 upon mitochondrial depolarization leads to the phosphorylation of a small fraction of RAB7a on S72, in a manner that is dependent on PINK1-Parkin activity in cells (Figure 1). 

What is the biochemical consequence of RAB7a S72 phosphorylation? The phosphomimetic RAB7a-S72E displayed reduced association with GDP dissociation inhibitor (GDI) proteins, as well as components of the Rab geranylgeranyltransferase (GGTase) complex. On the other hand, RAB7a-S72E displayed an enhanced association with a protein complex consisting of folliculin and the folliculin-interacting protein 1 (FLCN-FNIP1). FLCN and FNIP1 are Differentially expressed in neoplastic versus normal cells (DENN) domain-containing proteins [106] with potential GEF or GAP activities towards small GTPases [107,108], although neither activities of FLCN-FNIP1 could be confirmed for RAB7a. FLCN-FNIP1 could be recruited to damaged mitochondria and appears to promote Parkin-dependent mitophagy, and this recruitment is defective in cells with a non-phosphorylatable RAB7a-S72A mutant knocked-in. In fact, cells bearing RAB7a-S72A could no longer support efficient mitophagy, and exhibited a defect in the recruitment of ATG9a-positive vesicles to damaged mitochondria. This defect indicates that RAB7a’s phosphorylation at S72 is critical for its role in mitophagosome formation. However, cells lacking FLCN could still recruit ATG9a to damaged mitochondria and FLCN-FNIP1, thus this does not appear to be required for the RAB7a-dependent ATG9a recruitment. Notably, a subset of Rabs, including Rab8a, Rab8b, and Rab13, are known to be phosphorylated by PINK1 [109]. Whether Rab7a is also a substrate of PINK1 is unclear at the moment.

Regulation of Rab7a’s phophosphorylation status was shown to be important for late endocytic trafficking and signaling of epidermal growth factor receptor [94], as PTEN’s dephosphorylation of Rab7a on both S72 and Y183 is necessary for GDI-dependent recruitment of Rab7a to late endosomes. PTEN is an important positive regulator of autophagy [110] through its suppression of phosphatidylinositol 3-kinase/Akt kinase signaling and the loss of PTEN inhibits autophagy [111]. The findings of Heo and colleagues is particularly interesting when placed beside the perspectives of two other recent reports on the role of PTEN in mitophagy. Li and colleagues have recently shown that PTENα interacts with Parkin and promotes the latter’s recruitment to damaged mitochondria [96]. On the other hand, Wang and colleagues found that PTEN-long (PTEN-L) (which is PTENα) acts as a negative regulator of mitophagy by dephosphorylating phosphorylated ubiquitin [95]. It would be interesting to see which of the PTEN isoforms, α and β, could dephosphorylate RAB7a at S72 and modulate the latter’s role in mitophagosome formation. 

## 6. New Perspectives and Unanswered Questions

The recent findings pertaining to Rab7a’s role in mitophagy discussed above have generated a fairly novel notion. RAB7a, other than its role in autophagosome–lysosome fusion, could also function at the early stages of autophagy, at least in the case of Parkin-dependent mitophagosome formation during mitochondrial damage. This activity of Rab7a is dependent on its recruitment to, and proper regulation of its Rab cycle at the mitochondrial membrane. A tentative mechanism whereby Rab7a could drive mitophagosome formation is through the transport of Atg9a containing membranes to the forming and expanding phagophore. 

Many questions arise from this notion above, and two of the most pressing ones shall be briefly pondered upon here. Firstly, is the role of Rab7a specific for mitophagy and not for other forms of autophagy? Current data suggest that the ubiquitination of mitochondrial outer membrane proteins by Parkin, which occurs specifically on damaged mitochondria, presents a tentative mechanism to recruit Rab7a that does not occur prominently in, for example, starvation-induced autophagy. However, whether this Rab7a requirement is specific for mitophagosome formation would require further investigations. 

Secondly, although it is conceivable that mitophagy may rely more heavily on membrane input from ATG9a-bearing membranes and vesicles than starvation-induced autophagy, the mechanism of how Rab7a drives phagophore/IM extension around damaged mitochondria is unclear. The mechanism of inter-membrane transport of ATG9a and its associated membranes are poorly understood. Interestingly, recent findings in the fungal pathogen, *Fusarium graminearum*, may shed some light in this regard. The *Fusarium* Atg9 orthlogue, FgAtg9, is localized to the late endosomes and TGN and exhibits dynamic actin-dependent trafficking, which is regulated by the Rab7a orthologue, FgRab7. This dynamic FgAtg9 trafficking appears to be essential for autophagy-dependent development and the pathogenicity of the fungus in plants. Co-immunoprecipitation analyses showed that FgAtg9 associates with FgRab7 in vivo. If this interaction is confirmed and found to be conserved in mammalian cells, RAB7a at the damaged mitochondria could potentially facilitate the recruitment of ATG9-containing membrane vesicles through the interaction. Potential RAB7a–ATG9a interaction could also be involved in the engagement of tethering complexes that would facilitate ATG9a membrane docking onto the growing phagophore, or the regulation of the SNARE-mediated fusion process.

From the perspective of neurological diseases, RAB7a missense mutations underlie the inherited peripheral neuropathy, Charcot-Marie-Tooth type 2B (CMT2B) [112], while TBK1 mutations are associated with ALS [113]. Autophagy and mitophagy defects are well documented in ALS [114] and autophagic flux was recently found to be perturbed in cells from a CMT2B patient with an RAB7a-V162M mutation [115]. How RAB7a mutations affect mitophagy in CMT2B patients’ neurons is yet unclear, but the relevance of the notion of RAB7a having a role in mitophagosome formation to disease conditions is be worthy of further investigations. This is particularly so in view of the findings that mitophagy is a neuroprotective process and mitigates the pathology of major neurodegenerative diseases, such as Alzheimer’s disease [116] and Huntington’s disease [117].

Finally, it should be noted that the Rab7a kinase identified by Heo et al. [97], TBK1, is a key component [118] of the cyclic GMP-AMP synthase (cGAS)-Stimulator of IFN genes (STING) pathway of the innate immune response [119], which is linked to inflammatory pathologies, including neuroinflammation [120]. Defects in PINK1- and Parkin-mediated mitophagy likely contribute to Parkinson’s disease pathology in a profound manner, and recent work has indicated that functional PINK1-Parkin mitochondrial homeostasis may mitigate STING-mediated inflammation induced by exhaustive exercise or the accumulation of mitochondria DNA mutations [121]. This tantalizing link between inflammation and mitophagy and its potential role in neurodegeneration is worth pursuing in the immediate future.
